# Structural Characteristics, Binding Partners and Related Diseases of the Calponin Homology (CH) Domain

**DOI:** 10.3389/fcell.2020.00342

**Published:** 2020-05-14

**Authors:** Lei-Miao Yin, Michael Schnoor, Chang-Duk Jun

**Affiliations:** ^1^Laboratory of Molecular Biology, Shanghai Research Institute of Acupuncture and Meridian, Shanghai University of Traditional Chinese Medicine, Shanghai, China; ^2^Molecular Biomedicine, Center for Investigation and Advanced Studies of the National Polytechnic Institute (Cinvestav), Mexico City, Mexico; ^3^School of Life Sciences, Gwangju Institute of Science and Technology, Gwangju, South Korea

**Keywords:** actin cytoskeleton, CH-domain-containing proteins, α-helix, tubulin, calmodulin, tropomyosin, transgelin-2, cancer

## Abstract

The calponin homology (CH) domain is one of the most common modules in various actin-binding proteins and is characterized by an α-helical fold. The CH domain plays important regulatory roles in both cytoskeletal dynamics and signaling. The CH domain is required for stability and organization of the actin cytoskeleton, calcium mobilization and activation of downstream pathways. The CH domain has recently garnered increased attention due to its importance in the onset of different diseases, such as cancers and asthma. However, many roles of the CH domain in various protein functions and corresponding diseases are still unclear. Here, we review current knowledge about the structural features, interactome and related diseases of the CH domain.

## Introduction

**FIGURE 1:**
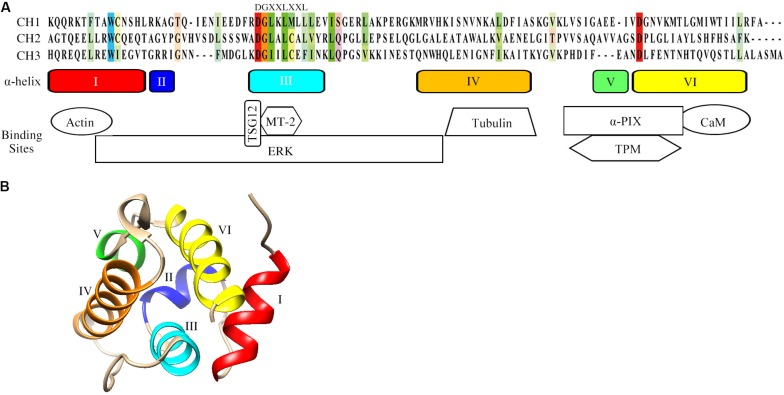
Structural characteristics of the CH domain. **(A)** Sequence alignment, schematic of the secondary structure elements and the binding sites of CH domain for actin and signaling proteins. The conserved residues among the three CH domains are colored. Schematics of the secondary structure elements of CH domain and binding sites are also included. UniProt identifiers for CH1 (α-actinin), CH2 (MICAL, Molecule interacting with CasL) and CH3 (calponin-1) are P12814, Q8TDZ2, P51911. **(B)** The tertiary fold of the calponin CH domain (PDB: 1H67). The CH domain contains in total six α-helices. Helices III and VI are approximately parallel, while helix IV is lying oblique aside. The structural model was generated by UCSF Chimera. Abbreviations: αPIX: Cdc42/Rac1-specific guanine nucleotide exchanging factor; CaM: calmodulin; ERK: extracellular signal-regulated kinase; MT-2: metallothionein-2; TPM: tropomyosin; TSG12: a specific transgelin-2 agonist.

Actin is an essential cytoskeletal protein that plays a critical role in multiple cellular processes (Pollard and Goldman, 2018). Actin monomers are assembled into different filamentous structures to form the actin cytoskeleton, which is a highly dynamic structure that regulates many cell processes such as adhesion, spreading and migration. Actin cytoskeletal dynamics require the coordinated action of many different actin-binding proteins (ABPs) (Garcia-Ponce et al., 2015). Since the discovery of actin-binding proteins, such as α-actinin and filamin, filamin in the 1970s (Lazarides and Burridge, 1975; Shizuta et al., 1976), more than 160 different members have been identified (Lappalainen, 2016; Kuhn and Mannherz, 2017). Calponin is an ABP that is expressed in smooth muscle and multiple types of non-muscle cells (Liu and Jin, 2016). The calponin homology (CH) domain, first identified at the N-terminus of calponin, is a common peptide module of approximately 100 residues and its precise number varies from protein to protein (Castresana and Saraste, 1995; Korenbaum and Rivero, 2002; Gimona and Winder, 2008). Sequence alignment of the CH domain shows that the residues of tryptophan (W) in helix I and aspartate (D) in helix VI are the most-conserved residues, while the consensus motif DGXXLXXL appears in helix III (Figure 1A; Gimona and Winder, 2008). The CH domain has been identified in a variety of proteins (CH-domain-containing proteins, CCPs), whose functions range from actin cross-linking to signal regulation (Gimona and Winder, 2008). Three types of CH domains have been described mainly based on their functions (Gimona et al., 2002; Korenbaum and Rivero, 2002). The type 1 CH domain (CH1) has the intrinsic ability to bind to F-actin. The type 2 CH (CH2) domain binds in tandem with CH1 and is required to facilitate high-affinity binding of F-actin. The type 3 CH domain (CH3) usually acts as a single CH domain in several ABPs and signaling proteins. Despite similarity of the secondary structure with the other types of CH domains, the CH3 domain shows functional diversity due to its ability to interact with many different proteins (Stradal et al., 1998).

Recent studies have reported that CCPs, including molecule interacting with CasL (MICAL), leucine-rich repeats and calponin homology containing 4 (Lrch4), smoothelin-like 1 (SMTNL1) and transgelin-2, exhibit an unappreciated functional variety and play important roles in the onset of various diseases (Table 1; Yin et al., 2018; Min et al., 2019). However, the precise role of how the CH domain confers various protein functions to drive such diseases is still unclear. In this review, we summarize the structural features, binding partners and diseases related to the CH domain.

**TABLE 1:** CH-domain-containing proteins involved in the onset and progression of different diseases.

Name	Domain type	Main expression	Related diseases	References
α-actinin	CH1-CH2	Muscle cells	Glomerulosclerosis congenital macrothrombocytopenia, hypertrophic cardiomyopathy	Mills et al., 2001; Haywood et al., 2016; Murphy et al., 2016
β-III-Spectrin	CH1-CH2	Purkinje cells, dendritic cells	Spinocerebellar ataxia	Ikeda et al., 2006; Avery et al., 2017b
Dystrophin	CH1-CH2	Muscle cells	Muscular dystrophy	Dumont et al., 2015
Filamin A	CH1-CH2	Nervous system, muscle cells	Otopalatodigital syndromes, cardiac valve disease, breast cancer	Norris et al., 2010; Tian et al., 2013; Lah et al., 2016
SMTNL1	CH2	Smooth muscle tissues	Cerebral arteriovenous malformations	Ulke-Lemee et al., 2011
MICAL	CH2	Nervous system	Spinal cord injury, breast cancer, kidney cancer	Pasterkamp et al., 2006; Deng et al., 2018
Calponin-1	CH3	Smooth muscle cells	Hypertension, breast cancer	Blascke de Mello et al., 2019; Wang et al., 2020
Lrch4	CH3	Spleen and thymus	Infection	Jha et al., 2010
Transgelin-2	CH3	Smooth muscle cells, immune cells	Asthma, systemic lupus erythematosus	Kiso et al., 2017; Yin et al., 2018

*CH, calponin homology; Lrch4, leucine-rich repeats and calponin homology containing 4; MICAL, molecule interacting with CasL; SMTNL1, smoothelin-like 1.*

## The Structure of the CH Domain

Understanding common CH domain structural features can help to elucidate the functions of CCPs. The CH domain is mainly α-helical and the strictly conserved residues in α-helices constitute an invariant hydrophobic core (Korenbaum and Rivero, 2002). The tertiary structure of the CH domain is compact and maintained by a network of hydrophobic interactions (Bramham et al., 2002). The CH domain contains six α-helices in total, including a core of four α-helices (I, III, IV, and VI) and two short helical structures (II and V, Figure 1A). A 3_10_-helical turn is also present in the loop between helices IV and V. Three helices of the CH domain of calponins (III, IV, and VI) form a triple-helix bundle, and helix I binds at a right angle across the surface provided by helices III and VI (Figure 1B). Unlike the majority of the CH domains, which are generally located at the N-terminus of proteins, the single CH2 domain of SMTNL1 is at its C-terminus. Nuclear magnetic resonance data show that the CH2 domain of SMTNL1 adopts the same α-helical fold as other CH domains and the most notable difference is the “KTKKK” cluster in the final helix (Ishida et al., 2008). The cluster “KTKKK” is unique in SMTNL1 and may be the potential site for ubiquitinylation (Ishida et al., 2008).

## Functional Regulation of the CH Domain

Changes in the linker and flanking regions of the CH domain regulate the configuration of the domains, thus influencing functional regulation and affinity for their interaction partners. A hinge (GLQQTN) in the linker region between the CH1-CH2 domain of dystrophin acts like a swivel to allow these conformational transitions (Fealey et al., 2018). After the linkers of the dystrophin and utrophin tandem CH domains were swapped, the dystrophin tandem CH domain with an utrophin linker (DUL) showed a 2-fold higher binding affinity compared to that of the dystrophin tandem CH domain, while the utrophin tandem CH domain with a dystrophin linker (UDL) had a 50% lower binding affinity than the utrophin tandem CH domain (Bandi et al., 2015). A chimera containing the CH1-CH2 domain from utrophin and the linker region from filamin A had a significantly higher actin-binding affinity (*K*_D_ = 0.7 μM) than wild-type utrophin (*K*_D_ = 19 μM) (Harris et al., 2019). Moreover, the N-terminal flanking region of the CH domain influences the binding of F-actin. After truncation of a flanking region of utrophin (MAKYGEHEASPDNGQNEFSDIIKSRSD), the binding affinity for F-actin was significantly decreased by approximately 2-fold in HeLa and HEK293T cells (Harris et al., 2019). However, *in vitro* co-sedimentation assays (not in live cells) showed that the same truncated utrophin binds to F-actin 30 times weaker than the full-length protein (Singh et al., 2017).

## Binding Partners of the CH Domain

The functional diversity of different CH domains is a result of different binding partners including actin, tubulin and signaling proteins as described below (Galkin et al., 2006).

### Binding With Actin and Tubulin

The exact mechanisms regulating binding between actin and the CH domain are still unclear. This process is associated with complex factors, including the CH domain number (single or a tandem pair), conformational differences, and flanking sequences (Galkin et al., 2010; Harris et al., 2019). The CH domain mainly acts in tandem pairs for F-actin binding (Sjoblom et al., 2008). Cryo-electron microscopy data revealed that the CH1 domain of human filamin A contributed to F-actin binding without direct CH2 and actin interactions (Iwamoto et al., 2018). Binding of the CH1-CH2 domain and actin is mechanically regulated via closed or open conformations (Shams et al., 2016). The CH1 domain contains the main actin-binding sites, however, one of the binding sites of CH1 is buried within the CH1-CH2 interface and only becomes accessible in the open conformation (Borrego-Diaz et al., 2006). Therefore, the CH2 domain serves as a locator domain to position the true actin-binding motifs, including the regulation of CH1 binding with actin, prevention of actin clashes and stabilization of the actin-binding domain (Galkin et al., 2006; Iwamoto et al., 2018). Co-sedimentation assays showed that the binding to F-actin by a single CH1 domain of human utrophin was about 5-fold weaker than that of the CH tandem pair, while both binding constants were 1000-fold stronger than that of the single CH2 domain (Singh et al., 2014).

Residue mutations can also affect binding between the CH domain and F-actin. For example, the CH2 domain mutation (L253P) of β-III-spectrin caused opening of the CH1-CH2 domains and promoted the N-terminal region of CH1 to become α-helical, thus enhancing approximately 1000-fold the actin-binding affinity (Avery et al., 2017a). In contrast, a single cysteine mutation (C10S before the CH1 domain or C188S in the middle of the CH2 domain) did not affect the structure or stability of the CH1-CH2 domain of dystrophin (Singh and Mallela, 2012). However, the K237E mutation in CH2 of α-actinin decreased the open conformation strength of the CH domain and increased actin-binding affinity (Shams et al., 2016).

Whether a single CH domain binds actin is still controversial. By constructing calponin without C-terminal tandem repeats, the resulting protein with the CH domain failed to bind to actin (Gimona and Mital, 1998), suggesting that a single CH domain is neither sufficient nor necessary for the binding of F-actin (Gimona and Mital, 1998). However, new findings showed opposite results within cells. Transgelin-1 interacted with actin via its CH3 domain, while the C-terminal tandem repeats were dispensable for actin-binding in smooth muscle cells (Matsui et al., 2018). While the wild-type transgelin-1 or transgelin-1 without the C-terminal tandem repeats both displayed fibrous patterns, the truncated protein with deletion of the CH3 domain showed diffuse patterns after separate transfection into A7r5 smooth muscle cells (Matsui et al., 2018).

The CH domain can also bind other cytoskeletal proteins such as tubulin. The end-binding protein 1 (EB1) is the first example of a single CH domain that can associate with tubulin (Hayashi and Ikura, 2003). A truncated version of EB1 containing only the CH domain co-sedimented with tubulin (Hayashi and Ikura, 2003). In contrast, the mutation K89E within α-helix IV, close to the hydrophobic cleft of the CH domain, abolished tubulin binding (Hayashi and Ikura, 2003). Deletion of the N-terminal 207 amino acid region of Hec1 (i.e., Hec1 without CH domain) resulted in failure of chromosomes alignment at the spindle equator during mitosis in PtK1 cells (Guimaraes et al., 2008). By contrast, deletion of only the N-terminal 80 amino acid tail of Hec1 (i.e., Hec1 with the CH domain) did not affect protein function. These findings together suggested that the CH domain of Hec1 is required for efficient binding of tubulin (Guimaraes et al., 2008). These new evidences clearly show the distinctive binding mechanisms of the CH domains with actin and tubulin, highlighting the need for further investigation into the functional mechanisms of these binding patterns.

### Binding With Signaling Proteins

Besides its ability to bind to actin and tubulin, the CH domain can participate in signal transduction by binding to different protein partners such as extracellular regulated kinase (ERK) and calmodulin (Figure 1A). The CH domain of calponin was identified as the binding site for ERK by sequencing chymotryptic fragments of calponin (Leinweber et al., 1999). Calponin thus facilitates the formation of signaling complexes with ERK and other kinases, such as protein kinase C (Leinweber et al., 2000). SMTNL1 can also interact with signaling proteins, including calmodulin and tropomyosin. The sequence IQELYRSLVQK in the α-helix VI of the SMTNL1 CH2 domain is the binding site for calmodulin and the K_D_ obtained by isothermal titration calorimetry was 2.7 × 10^–6^ M (Ishida et al., 2008). SMTNL1 can be phosphorylated by protein kinase A (PKA) at Ser301, which lies upstream of the CH domain, and this phosphorylation strongly enhances the ability of SMTNL1 to associate with tropomyosin (Ulke-Lemee et al., 2017). However, the exact binding region between SMTNL1 and tropomyosin that may affect the modulation of muscle contractile activity is still uncertain (Ulke-Lemee et al., 2010). Removal of the CH2 domain or expression of the CH2 domain of SMTNL1 alone did not enable binding with tropomyosin, suggesting that the CH2 domain is not sufficient to mediate binding but is involved in the regulation of the binding affinity for tropomyosin (Ulke-Lemee et al., 2010). However, the study further shows that a portion of the N-terminal intrinsically disordered region (1–341 residues) of SMTNL1 forms intramolecular contacts with its C-terminal CH domain, SMTNL1 thus interacts with tropomyosin at residues 421–436, which encompasses the entirety of α-helix V and the beginning of α-helix VI of the CH2 domain (MacDonald et al., 2012).

For other CCPs, the N-terminal 53–271 residues of affixin that cover the CH1 domain but not the CH2 domain are the binding sites of Cdc42/Rac1-specific guanine nucleotide exchanging factor (αPIX), as shown using co-immunoprecipitation assays (Mishima et al., 2004). Transgelin-2 with a CH3 domain is a receptor for extracellular ligands such as metallothionein-2 (Crunkhorn, 2018; Yin et al., 2019). The small compound TSG12, which was identified through molecular docking by targeting 46–63 residues of the CH3 domain of human transgelin-2 (QPGRENFQKWLKDGTVLC) induced dephosphorylation of myosin phosphatase-targeting subunit 1 (MYPT1) (Yin et al., 2018).

## The Role of CH Domains in Various Diseases

CCPs, including MICAL1/2, Lrch4 and SMTNL1, have been shown to play crucial roles in various diseases as discussed below. A summary of the involvement of CH domains in various diseases is shown in Table 1.

### MICAL1/2

MICAL1/2 contains a CH2 domain and oxidizes methionine residues of actin to disassemble F-actin into G-actin (Grintsevich et al., 2016). The MICAL1/2 CH2 domain is connected to the monooxygenase domain, and Arg530 in the CH2 domain is the key residue mediating interaction with the monooxygenase domain (Kim et al., 2020). MICAL1/2 can also regulate actin dynamics and cell morphological changes via the CH2 domain through interacting with other signaling proteins (Hung et al., 2010).

Studies have shown that MICAL proteins are closely related to neural diseases and cancers. MICAL expression is substantially elevated in oligodendrocytes and in meningeal fibroblasts during spinal cord injury, suggesting an involvement of MICAL in neuronal regeneration (Pasterkamp et al., 2006). Targeting MICAL may provide a new therapeutic option for cancer treatment (Yoon and Terman, 2018). For example, deletion of MICAL1 substantially reduced cell proliferation in the breast cancer cell lines MCF-7 and T47D (Deng et al., 2018). Over-expression of MICAL2 in MCF-7 cells augmented the level of epidermal growth factor receptor (EGFR) in the plasma membrane, thus enhancing cell migration (Wang et al., 2018). In contrast, silencing MICAL2 in MDA-MB-231 cells degraded EGFR and inhibited cell migration (Wang et al., 2018). MICAL2 gene expression was significantly increased in aggressive primary gastric and renal cancers (Mariotti et al., 2016). MICAL2 knockdown caused a reduction in viability and loss of motility and invasion in 786-O kidney cancer cells, suggesting that MICAL2 might be a promising target for anti-metastatic therapy (Mariotti et al., 2016).

### Lrch4

Lrch4 is a plasma membrane protein abundantly expressed in the spleen and thymus, containing a single-pass transmembrane domain with nine leucine-rich repeats and a CH3 domain in its ectodomain (Aloor et al., 2019). Recent data showed that Lrch4 did not interact with ezrin, radixin and moesin (ERM) in drosophila S2 cells, suggesting that the CH3 domain of Lrch4 may not bind with the FERM domain of ERM (Foussard et al., 2010). The function of the CH3 domain in Lrch4 is still unclear (Aloor et al., 2019).

Lrch4 is a novel Toll-like receptor (TLR) accessory protein as Lrch4 knockdown attenuated TNFα secretion induced by various TLR ligands (Aloor et al., 2019). Therefore, Lrch4 has been considered a broad-spanning regulator of the innate immune response and a potential molecular target in inflammatory diseases (Aloor et al., 2019). Lrch4 was identified by mass spectrometry to be differentially expressed in macrophages 24 h after infection with *Mycobacterium avium* subsp *hominissuis* (Jha et al., 2010). Microarray analysis showed that the gene expression of Lrch4 was up-regulated by 1.6-fold in lipopolysaccharide-stimulated dendritic cells in inflammation (Ceppi et al., 2009).

### SMTNL1

Smoothelin-like 1, which contains a CH2 domain in the C-terminal region, is a novel member of the smoothelin protein family (Borman et al., 2004). Deletion of the CH2 domain can significantly change the intracellular localization of SMTNL1 (from distributed longitudinally on F-actin to diffuse distribution in the cytoplasm) in rat vascular smooth muscle cells suggesting that the CH2 domain is critical for F-actin binding (Ulke-Lemee et al., 2010). Experiments with truncated recombinant proteins showed that the CH2 domain was essential for SMTNL1-associated smooth muscle relaxation because the CH2 domain alone did not cause relaxation in rabbit ileum smooth muscle strips (Borman et al., 2009).

Smoothelin-like 1 can modulate muscle contractility, and its biological activity may involve interaction with the contractile actin machinery (Ulke-Lemee et al., 2010). One of the target genes of SMTNL1 is MYPT1 (high expression of MYPT1 is associated with the contraction of smooth muscle), and SMTNL1 knock-out increased MYPT1 protein expression by 30- to 40-fold in neonatal mice (Lontay et al., 2010). SMTNL1 interacts with myosin phosphatase in the cytoplasm, however, when phosphorylated at Ser301 in response to PKA/PKC, SMTNL1 translocates into the nucleus where it may activate transcription factors driving MYPT1 expression (Lontay et al., 2010). The gene expression of SMTNL1 was also significantly increased by approximately 4-fold in human cerebral arteriovenous malformations, suggesting that the elevated level of SMTNL1 may decrease MYPT1 expression to relax brain blood vessels and thus contribute to this lumen disorder (Yao et al., 2019).

## Conclusion and Prospects

The CH domain displays high structural conservation, but shows diverse biological functions. The indispensable flanking regions and/or intrinsically unfolded protein modules may contribute to orchestrating CH domain functions. However, when comparing cell-based experiments with *in vitro* experiments using recombinant proteins only, it should be noted that due to the complexities of the cellular environment, other factors could be coming into play and distorting the results. With newly discovered proteins that interact with CH domains, some of the diverse functions have now been elucidated. However, many other binding proteins and functions certainly remain to be discovered, thus warranting further research into CH domain biology. Given that numerous CCPs, such as MICAL and transgelin-2, have been identified as promising therapeutic targets in diseases, it will be important to investigate in the future whether compounds can be designed to specifically target CH domains and thus improve the outcome of certain diseases.

## Author Contributions

L-MY designed the work, wrote the manuscript, and prepared the figures. MS and C-DJ drafted and revised the manuscript. All authors contributed to manuscript revision, read and approved the submitted version.

## Conflict of Interest

The authors declare that the research was conducted in the absence of any commercial or financial relationships that could be construed as a potential conflict of interest.
